# Molecular characterization, ultrastructure, and transovarial transmission of *Tremblaya phenacola* in six mealybugs of the Phenacoccinae subfamily (Insecta, Hemiptera, Coccomorpha)

**DOI:** 10.1007/s00709-019-01405-y

**Published:** 2019-06-27

**Authors:** Anna Michalik, Katarzyna Michalik, Beata Grzywacz, Małgorzata Kalandyk-Kołodziejczyk, Teresa Szklarzewicz

**Affiliations:** 1 Department of Developmental Biology and Morphology of Invertebrates, Institute of Zoology and Biomedical Research, Faculty of Biology, Jagiellonian University, Gronostajowa 9, 30-387 Kraków, Poland; 2 Institute of Systematics and Evolution of Animals, Polish Academy of Sciences, Sławkowska 17, 31-016 Kraków, Poland; 3 Department of Zoology, Faculty of Biology and Environmental Protection, University of Silesia, Bankowa 9, 40-007 Katowice, Poland

**Keywords:** Mealybugs, *Tremblaya phenacola*, Bacteriocyte, Transovarial transmission

## Abstract

Mealybugs (Hemiptera, Coccomorpha: Pseudococcidae) are plant sap-sucking insects which require close association with nutritional microorganisms for their proper development and reproduction. Here, we present the results of histological, ultrastructural, and molecular analyses of symbiotic systems of six mealybugs belonging to the Phenacoccinae subfamily: *Phenacoccus aceris*, *Rhodania porifera*, *Coccura comari*, *Mirococcus clarus*, *Peliococcus calluneti*, and *Ceroputo pilosellae*. Molecular analyses based on bacterial 16S rRNA genes have revealed that all the investigated species of Phenacoccinae are host to only one type of symbiotic bacteria—a large pleomorphic betaproteobacteria—*Tremblaya phenacola*. In all the species examined, bacteria are localized in the specialized cells of the host-insect termed bacteriocytes and are transovarially transmitted between generations. The mode of transovarial transmission is similar in all of the species investigated. Infection takes place in the neck region of the ovariole, between the tropharium and vitellarium. The co-phylogeny between mealybugs and bacteria *Tremblaya* has been also analyzed.

**Electronic supplementary material:**

The online version of this article (10.1007/s00709-019-01405-y) contains supplementary material, which is available to authorized users.

## Introduction

The existence of many organisms depends on the presence of obligatory symbionts. This association is usually nutritional in character. The symbionts of hemipterans which feed on phloem or xylem sap provide them amino acids absent in their diet which they cannot synthesize de novo (Douglas 1998; Dale and Moran 2006; McCutcheon and Moran 2007; Douglas 2016). In turn, blood-feeding insects, such as lice and Hippoboscoidae flies receive B vitamins which are lacking in vertebrate blood from their symbiotic partner (Chen et al. 1981; Akman et al. 2002; Moriyama et al. 2015; Meseguer et al. 2017). In some insects, symbionts may also be engaged in the production of enzymes and co-factors, as well as play an important role in the recycling of uric acid (Chen et al. 1981; Sasaki et al. 1996; Patiño-Navarrete et al. 2014).

Some hemipterans require multi-partner association, in which all members of symbiotic consortium contribute to the synthesis of all nutrients necessary for the host-insect (review: Douglas 2016). Symbionts which supplement each other with respect to provisioning amino acids to the host-insect, as well as other elements missing in their diet, were termed “co-primary symbionts” (Takiya et al. 2006). The term “co-primary symbionts” was first used by Takiya et al. (2006) for the symbiotic systems of Hemiptera: Auchenorrhyncha (cicadas, leafhoppers, treehoppers, spittlebugs, and planthoppers) which usually harbor two obligate symbionts: bacterium *Sulcia* and one type of proteobacteria, e.g., alphaproteobacterium *Hodgkinia* in cicadas, betaproteobacterium *Nasuia* in Deltocephalinae leafhoppers, and gammaproteobacterium *Baumannia* in some Cicadellinae leafhoppers (Bennett and Moran 2013; Ishii et al. 2013; Bennett et al. 2014; Campbell et al. 2015; Kobiałka et al. 2016, 2018; Łukasik et al. 2018). A symbiotic system consisting of two microorganisms also occurs in many representatives of Sternorrhyncha such as aphids, psyllids, and whiteflies (Baumann 2005). These insects usually possess one type of obligate symbiont (e.g., *Buchnera* in aphids, *Portiera* in whiteflies, *Carsonella* in psyllids) which is associated with an additional symbiotic microorganism (Baumann 2005). Within symbiotic associates of Sternorrhyncha, Buchner (1965) distinguished primary symbionts (later termed P-symbionts) and accessory symbionts (later termed secondary symbionts, S-symbionts, facultative symbionts). Since recent genomic analyses indicate that, similarly to the co-primary symbionts in Auchenorrhyncha, the primary and secondary symbionts of Sternorrhyncha complement one another in synthesis of nutrients, they may be regarded as co-primary symbionts (Pérez-Brocal et al. 2006; Gosalbes et al. 2008; Luan et al. 2015). In contrast to aphids, whiteflies, and psyllids, scale insects are characterized by a great diversity of symbionts (Fukatsu and Nikoh 2000; von Dohlen et al. 2001; Thao et al. 2002; Baumann and Baumann 2005; Szklarzewicz et al. 2006, 2018; Gruwell et al. 2007, 2010; Niżnik and Szklarzewicz 2007; Matsuura et al. 2009; Rosenblueth et al. 2012; Husnik and McCutcheon 2016; Michalik et al. 2016; Michalik et al. 2018a). They may be host to two types of microorganisms which may exist as co-primary symbionts (Husnik and McCutcheon 2016; Szabo et al. 2017), or possess only one obligate symbiont (Gruwell et al. 2010, 2012; Michalik et al. 2016).

The neococcoid family Pseudococcidae (mealybugs) is the second most species-rich family within the infraorder Coccomorpha. It has been estimated to include over 1990 described species in 259 genera (García Morales et al. 2016). Pseudococcidae species feed on a wide variety of woody and herbaceous plants and are often restricted to a specific part of their host. Many of them are agricultural pests which have a damaging effect on the environment. They destroy plants not only by sucking their sap, but also by contaminating them with honeydew that serves as a substrate for sooty molds which impair photosynthesis. Additionally, scale insects may transmit plant viruses (Gullan and Cranston 2014).

The classification of the Pseudococcidae has been changed many times (Koteja 1974; Hardy et al. 2008), but at present, two subfamilies are recognized within this family: Phenacoccinae and Pseudococcinae (Hardy et al. 2008; Kaydan et al. 2015).

The symbionts of Pseudococcidae were first described by Walczuch (1932) and Buchner (1957, 1965). Buchner (1957, 1965) studied symbiosis in several mealybugs of the genera *Pseudococcus*, *Ferrisia*, *Trionymus*, and *Antonina* (all currently recognized as Pseudococcinae). Based on histological observations, Buchner (1957, 1965) stated that all the Pseudococcinae examined possess the same symbiosis type; however, the symbionts may differ in shape, both in various species and even within one bacteriocyte of the same individual. Walczuch (1932) and Buchner (1957, 1965) observed significant differences in the organization of the symbiotic systems in Pseudococcinae mealybugs and members of the genera *Phenacoccus* and *Rastrococcus* (both currently recognized as Phenacoccinae). More recent molecular analyses, including genome sequencing allowed for the determination of the systematic affinity and function of microorganisms associated with mealybugs (von Dohlen et al. 2001; Gruwell et al. 2010; McCutcheon and von Dohlen 2011; Koga et al. 2013; Lopez-Madrigal et al. 2014, 2015; Husnik and McCutcheon 2016; Szabo et al. 2017; Gil et al. 2018). Most of these studies concerned symbionts of representatives of the Pseudococcinae subfamily. As a rule, these mealybugs possess two types of bacterial symbiont: betaproteobacterium *Tremblaya princeps* and gammaproteobacterium. Both symbionts create a nested symbiotic consortium due to the fact that gammaproteobacterium always occurs in the cytoplasm of the *T. princeps* cells (von Dohlen et al. 2001; McCutcheon and von Dohlen 2011; Husnik et al. 2013; Husnik and McCutcheon 2016; Szabo et al. 2017). Molecular investigations involving the genome sequencing of the symbiotic systems of several Pseudococcinae mealybugs have revealed that the “*Tremblaya*-gammaproteobacterium” consortia function as a “metabolic patchwork.” Similarly to other long-term-associated co-primary symbionts, the bacterium *T. princeps* has an extremely reduced genome and does not possess complete pathways for essential amino acid biosynthesis (McCutcheon and von Dohlen 2011; Husnik and McCutcheon 2016). The genomes of gammaproteobacteria (related to the bacteria *Sodalis*) residing inside its cells are 3 to 4 times larger than the genome of *T. princeps.* In consequence, these bacteria complement *T. princeps* with respect to the products of the genes missing in its genome (McCutcheon and von Dohlen 2011; Husnik and McCutcheon 2016).

Symbiotic systems in the mealybugs belonging to the Phenacoccinae subfamily are less known. Gruwell et al. (2010) analyzed using molecular methods symbionts of 23 species of Phenacoccinae. Based on 16S rRNA gene sequences of their symbionts, these authors revealed that Phenacoccinae mealybugs (except genus *Rastrococcus*) are associated with betaproteobacterium *Tremblaya phenacola*. So far, the only genomic data available are for two *T. phenacola* genomes: *T. phenacola*—symbiont of *Phenacoccus peruvianus* and *T. phenacola* of *Phenacoccus avenae* (Husnik et al. 2013; Gil et al. 2018). These studies indicate that *T. phenacola*, as a single symbiont of Phenacoccinae mealybugs, provides all essential amino acids and vitamins to its host-insect.

Interestingly, Gil et al. (2018) have shown the chimeric nature of *T. phenacola* from *Phenacoccus peruvianus*—they found both beta—and gammaproteobacterial DNA in its genome. According to Lopez-Madrigal et al. (2014), the presence of gammaproteobacterial DNA in *T. phenacola* genome is a result of horizontal gene transfer between symbionts (i.e., betaproteobacteria and gammaproteobacteria) during the evolutionary history of this mealybug. Genome annotation and functional analysis allowed to state that chimeric genome of *T. phenacola* functions as a metabolic patchwork similar to that described for the dual symbioses in Pseudococcinae (Gil et al. 2018).

Taking into consideration the aforementioned data, the aim of the present paper was to describe the symbiotic systems of six representatives of the Phenacoccinae subfamily using molecular, histological, and ultrastructural approaches. Since there are only fragmentary data available on the morphology of *T. phenacola*, we described its distribution, ultrastructure, and mode of transovarial transmission. Additionally, the phylogenetic relationships of symbionts of mealybugs as well as the co-phylogeny between symbionts and their host-insects have been analyzed.

## Material and methods

### Insects

The symbiotic systems of six representatives of the Phenacoccinae subfamily were investigated: *Ceroputo pilosellae* Šulc, 1898, *Coccura comari* (Künow, 1880), *Mirococcus clarus***(**Borchsenius, 1949), *Phenacoccus aceris* (Signoret, 1875), *Rhodania porifera* Goux, 1935, and *Peliococcus calluneti* (Lindinger, 1912). The larvae and adult females of species examined were collected in Poland between the years 2012 and 2017. Localities, collection dates, and host plants of the investigated species have been summarized in Table 1.

**Table 1:** Localities, collection dates, and host plants of the investigated species

Species name	Date of collection	Host plant	Place of collection
*Coccura comari* (Künow, 1880)	06.2012 06.2014 07.2017	*Rubus* sp.	Ruda Śląska Rudy Wielkie Olsztyn near Częstochowa
*Ceroputo pilosellae* Šulc, 1898	05.2015; 07.2017	*Hieracium pilosella* L.	Twardowice Olsztyn near Częstochowa
*Rhodania porifera* Goux, 1935	10.2013; 09.2015; 04.2016; 05.2017; 09. 2017; 06.2017; 062017; 09.2017; 01.2018	Roots of *Festuca ovina* L.	Jaroszowiec Olkuski Olsztyn near Częstochowa Dąbrowa Górnicza, Sikorka
*Phenacoccus aceris* (Signoret, 1875)	04.2016; 05.2017	*Malus* sp.	Katowice
*Mirococcus clarus* (Borchsenius, 1949)	08.2017	*Festuca ovina* L.	Mikoszewo
*Peliococcus calluneti* (Lindinger, 1912)	08.2014	*Calluna vulgaris* (L.) Hull	Kalety

### Light and transmission electron microscopy

The larvae and adult females of each of the species investigated, destined for detailed histological and ultrastructural analyses, were fixed in 2.5% glutaraldehyde in 0.05 M phosphate buffer (pH 7.4), rinsed in the buffer with the sucrose (5.8 g/100 ml), postfixed in buffered 1% osmium tetroxide, dehydrated in an ethanol series (30%, 50%, 70%, 90%, 100%) and acetone, and finally embedded in epoxy resin Epon 812 (Serva, Germany). Semithin sections (1 μm thick) were stained in 1% methylene blue in 1% borax and photographed under a Nikon Eclipse 80i light microscope. The ultrathin sections (90 nm thick) were doubly contrasted with uranyl acetate and lead citrate and then examined and photographed under a Jeol JEM 2100 transmission electron microscope at 80 kV.

### Molecular analyses

The examined adult females and larvae of mealybugs destined for molecular analysis were fixed in 100% ethanol. DNA was then extracted separately from three individuals of each species using the Sherlock AX DNA extraction kit (A&A Biotechnology) following manufacturer’s protocol and subsequently stored at 4 °C for further analyses.

The 1.5 kb fragment of bacterial 16S rRNA gene sequence was amplified using universal, eubacterial primers: 10F and 1507R (Sandström et al. 2001) under the following conditions: an initial denaturation step at 94 °C for 3 min, followed by 33 cycles at 94 °C for 30 s, 55 °C for 40 s, 70 °C for 1 min 40 s, and a final extension step of 5 min at 72 °C. The PCR product was made visible by electrophoresis in 1.5% agarose gel stained with Simply Safe (Eurx) and following this, the PCR product was purified using a Clean-up purification kit (A&A Biotechnology). The purified PCR product was cloned into the pJET 1.2/blunt plasmid vector using Clone JET PCR Cloning Kit (Thermo Scientific). The ligated mixture was then transformed into competent *Escherichia coli* TOP10F cells which were subsequently prepared using the *E. coli* Transformer Kit (A&A Biotechnology). After 16 h, the occurrence of plasmid with bacterial 16S rRNA genes was confirmed by diagnostic colony PCR with the primers pJET1.2 Forward and pJET1.2 Reverse. The PCR reactions were performed according to the manufacturer’s protocol (https://www.thermofisher.com/order/catalog/product/K1231). Fifty positive colonies of each species examined were subjected to restrictive analysis using a *MspI* restrictive enzyme. The plasmids from the selected colonies were isolated using a Plasmid Mini AX kit (A&A Biotechnology) and following this, the representatives of each RFLP genotype were sequenced. The sequences obtained were then compared with other 16S rRNA gene sequences deposited in the GenBank database using BLAST.

Amplifications of COI genes of mealybugs analyzed were performed using PCR reactions with primers: PCoF1 and HCO (Hardy et al. 2008), under the following conditions: an initial denaturation step was performed at 94 °C for 3 min, followed by 35 cycles at 94 °C for 30 s, 51 °C for 40 s, 70 °C for 1 min 30 s, and a final extension step for 5 min at 72 °C.

PCR reactions of four additional genes encoding enzymes involved in the biosynthesis of essential amino acids in symbiotic systems of mealybugs: trpB (encoding the beta subunit of tryptophan synthase, involved in tryptophan biosynthesis), argH (encoding argininosuccinate lyase, involved in arginine biosynthesis), leuB (encoding 3-isopropylmalate dehydrogenase, involved in leucine biosynthesis), and metE (encoding cobalamin-independent homocysteine transmethylase, involved in methionine biosynthesis) were performed according to protocols provided by Lopez-Madrigal et al. (2014). Various PCR conditions including temperature gradient, different concentration of DNA template, and number of cycles have been tested.

The nucleotide sequences obtained were deposited in the GenBank database under the accession numbers MK159695-MK159700 and MK193743- MK193748.

### Fluorescence in situ hybridization

Fluorescence in situ hybridization (FISH) was conducted with a probe designed specifically for the 16S rRNA gene of Betaproteobacteria—BET940R: Cy5-TTAATCCACATCATCCACCG (Demanèche et al. 2008). Two individuals of each species preserved in 100% ethanol were rehydrated, fixed in 4% formaldehyde for 2 h, and dehydrated through incubation in 80%, 90%, and 100% ethanol and acetone. The material was then embedded in Technovit 8100 resin and subsequently cut into sections. Hybridization was performed using a hybridization buffer containing 1 ml 1 M Tris-HCl (pH 8.0), 9 ml 5 M NaCl, 25 μl 20% SDS, 15 ml 30% formamide, and about 15 ml of distilled water. The slides were incubated in 200 μl of hybridization solution (hybridization buffer + probe) overnight at room temperature (Łukasik et al. 2017). Next, the slides were washed in PBS three times for 10 min, then dried and covered with a ProLong Gold Antifade Reagent (Life Technologies). The hybridized slides were then examined using a confocal laser scanning microscope Zeiss Axio Observer LSM 710.

### Phylogenetic and co-phylogenetic analyses

Multiple alignments for COI sequences were made using the program CodonCode Aligner (CodonCode Corporation, www.codoncode.com, 2009) and coding regions were translated into amino acids in order to check for the presence of internal stop codons. Alignments for the bacterial 16S rRNA gene sequences were generated using Clustal X (Thompson et al. 1997). The genetic diversity (the number of polymorphic sites) was calculated using DnaSP v. 6 (Rozas et al. 2017). The phylogenetic signal was determined using PAUP* v. 4.0b10 (Swofford 2011). MrModeltest v. 2.2 (Nylander 2004) was used to perform a hierarchical likelihood ratio test and calculate the approximate AIC values of the nucleotide substitution models for each gene fragment. Bayesian inference (BI) was executed in MrBayes v. 3.2.6 (Ronquist et al. 2012), applying the parameters for the substitution model suggested by MrModeltest in each gene partition. Four Markov chains were run simultaneously for 10 million generations with sampling every 100 generations to ensure the independence of the samples. Two independent runs were performed to ensure that convergence on the same posterior distribution was reached and if final trees converged on the same topology. The statistical confidence in the nodes was evaluated using posterior probabilities. A maximum likelihood (ML) analysis was conducted under IQTree (Nguyen et al. 2015). Bootstrap percentages (BP) were computed after 1000 replicates.

Thirteen host species and their symbiotic bacteria *Tremblaya* were used for co-phylogeny. The sequences of the COI and 16S rRNA gene were employed in the analysis of the congruence of the symbiont and host phylogenies using the Jane v.4 (Conow et al. 2010). This software is a genetic algorithm which compares the two tree topologies by mapping the symbiont tree onto the host tree. The analysis was run with a default cost regime.

## Results

### Mealybugs of Phenacoccinae subfamily harbor one type of bacteriocyte-associated symbiont—betaproteobacterium *Tremblaya phenacola*

**Fig. 1:**
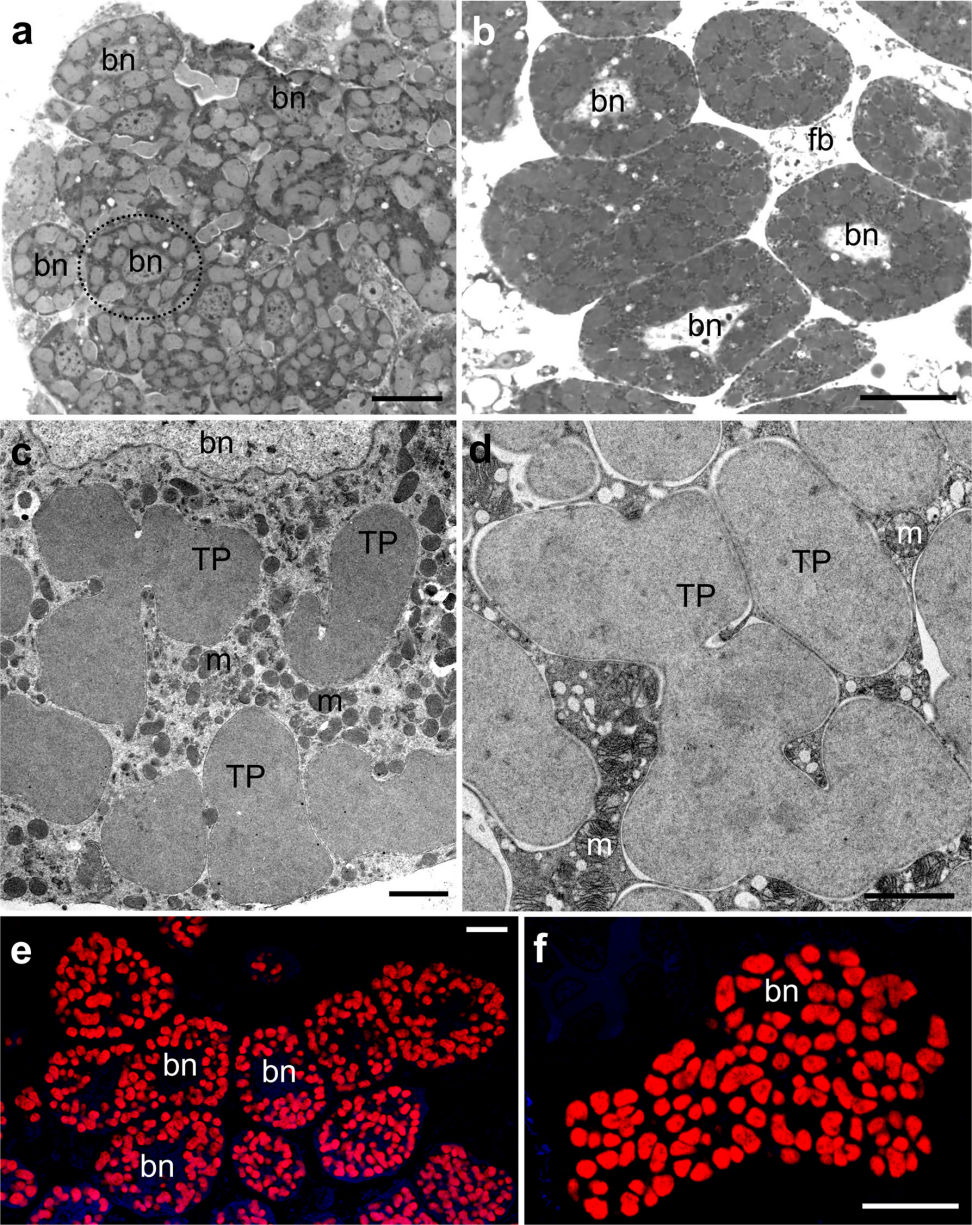
Distribution of symbionts in the body of Phenacoccinae mealybugs. **a***Coccura comari*. Fragment of the bacteriome consisting of several bacteriocytes (*encircled in black dotted line*) filled with symbiotic bacteria in the body cavity of young female. Bacteriocyte nucleus (*bn*). LM, scale bar = 20 μm. **b***C. comari*. Separated bacteriocytes in the body cavity of adult female. Bacteriocyte nucleus (*bn*); fat body (*fb*). LM, scale bar = 20 μm. **c**, **d** Fragment of the bacteriocyte with bacteria *T. phenacola* (*TP*). Note numerous mitochondria (*m*) in the cytoplasm of the bacteriocytes. Bacteriocyte nucleus (*bn*). **c***Phenacoccus aceris*, **d***Rhodania porifera*. TEM, scale bar = 2 μm. **e**, **f** Fluorescence in situ identification (FISH) of the bacteria *T. phenacola*. Bacteriocyte nucleus (*bn*). **e***P. aceris*, **f***Peliococcus calunetti*. Confocal microscope, scale bar = 20 μm

**Fig. 2:**
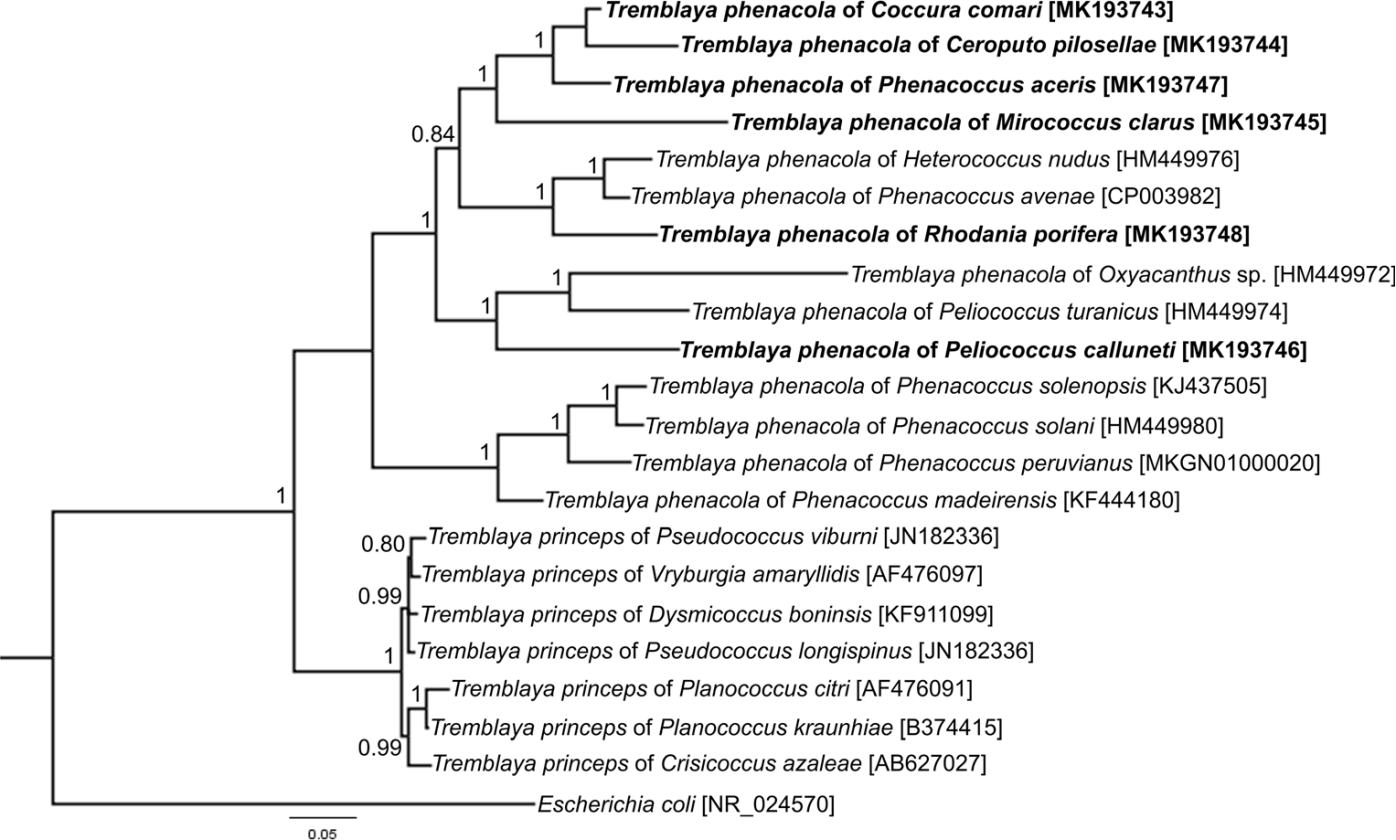
Phylogenetic relationships between bacteria *Tremblaya* based on the Bayesian analysis of their 16S rRNA gene sequences. The values on each branch represent the Bayesian posterior probability

Histological, ultrastructural, and molecular analyses have revealed that all of the species investigated are host to only one type of symbiotic microorganism (Figs. 1, 2, and S1). A comparison of 16S rRNA gene sequences of symbionts of mealybugs examined with data deposited in the GenBank database as well as phylogenetic analysis (Fig. 2) has shown that all of them harbor the betaproteobacterium *T. phenacola.* Since Gil et al. (2018) have shown the chimeric nature of *T. phenacola* associated with *Phenacoccus peruvianus* as well as Lopez-Madrigal et al. (2014) have found gammaproteobacterial DNA in the genome of *T. phenacola* of *Phenacoccus madeirensis*, we tried to amplify the following genes: trpB, argH, leuB, metE of *T. phenacola* from all Phenacoccinae species investigated. For this reason, we conducted PCR reactions with primers specific for genes mentioned above according to Lopez-Madrigal et al. (2014). Unfortunately, amplifications were unsuccessful for all genes despite numerous modifications of the protocols. Thus, to resolve the questions concerning the chimeric nature of *T. phenacola* of Phenacoccinae mealybugs examined in present study, the genome sequencing is needed.

In all the examined species of Phenacoccinae, symbiotic bacteria are localized in the specific insect cells termed bacteriocytes (Fig. 1a, b, and S1). Bacteriocytes are large, predominantly spherical cells which assemble into two large bacteriomes (Fig. 1a). The central part of each bacteriocyte is occupied by a large nucleus around which symbiotic bacteria are arranged (Fig. 1a, b). Ultrastructural observations have shown that like in other hemipterans in the bacteriocyte cytoplasm, numerous mitochondria are present (Fig. 1c, d). In all the species examined, bacteria *Tremblaya* are pleomorphic (Fig. 1c, d) and are surrounded by three membranes (not shown).

The systematic affiliation of bacterial symbionts residing in bacteriocytes in the examined species to Betaproteobacteria was confirmed by fluorescence in situ hybridization with a specific probe to betaproteobacterial symbionts (Fig. 1e, f).

### Bacteria *Tremblaya phenacola* constitute the monophyletic clade

The final DNA sequence dataset was comprised of 645 bp for COI and 1438 bp for 16S rRNA genes. The subfamily Phenacoccinae had around 38% of variable sites and 11% parsimony, informative for the COI and for 16S rRNA genes, the values were 36% and 27%, respectively. Every subset of molecular data revealed a strong phylogenetic signal (g1 < − 0.4, *p* < 0.001). MrModeltest identified the Sym + G model of substitution as the most appropriate for the COI and 16S rRNA gene dataset. The shapes of the phylogenies trees were roughly concordant among methods of reconstruction, although nodal support for ML outputs was typically lower than for BI.

A molecular phylogenetic comparison between the species of two Pseudococcidae subfamilies, along with their *T. princeps* and *T. phenacola* symbionts resulting from the Bayesian Inference (BI) analysis, is shown in Fig. 2. The maximum likelihood (ML) analysis yielded an almost identical topology (not shown). Within *T. phenacola*, the clade associated with Phenacoccinae hosts was recognized as monophyletic. 16S rRNA gene sequences of symbionts from the Phenacoccinae were sister to the 16S rRNA gene sequences of *T. princes* lineage.

**Fig. 3:**
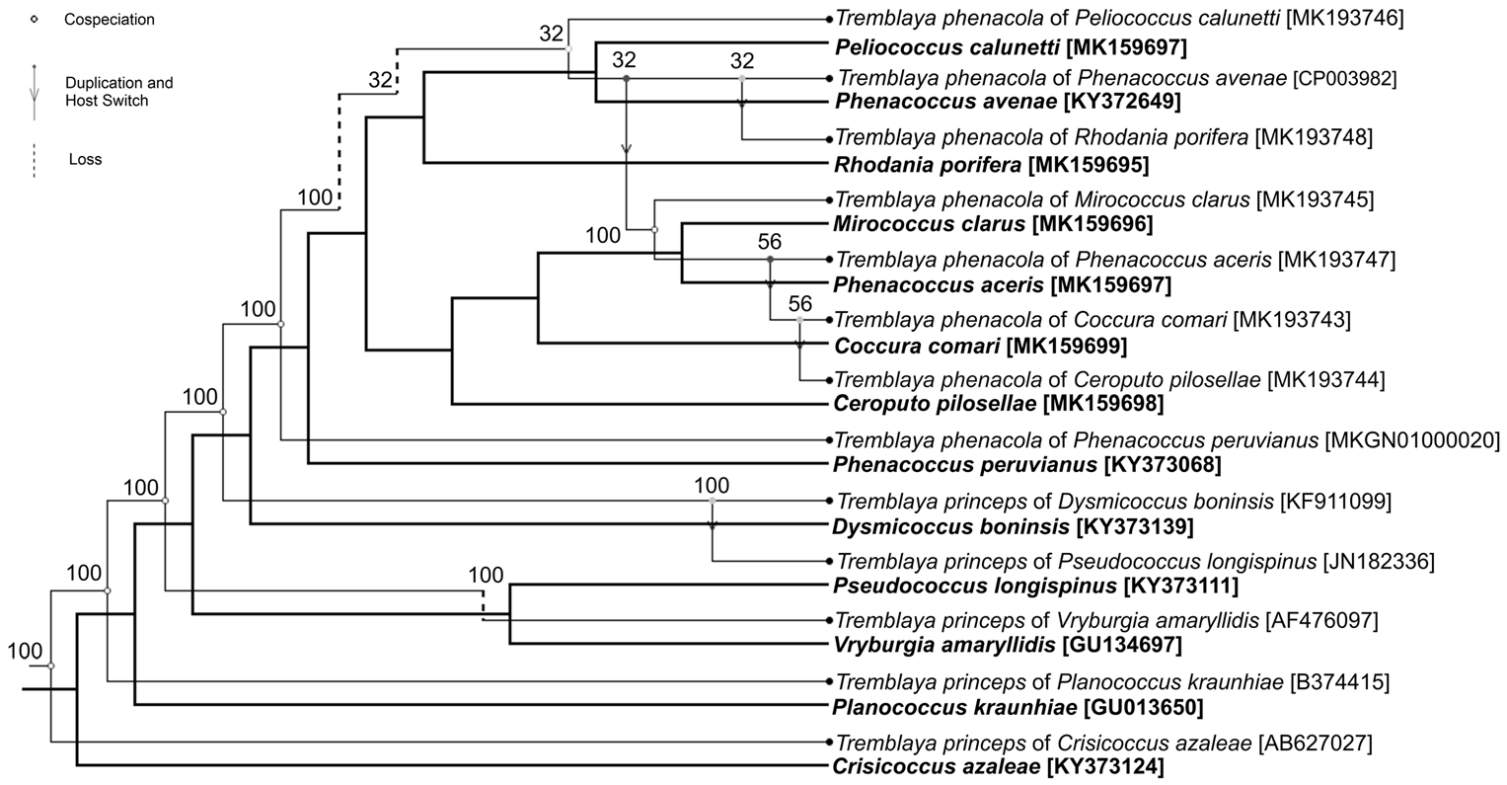
One of the 81 possible co-phylogenetic scenarios between the *Tremblaya* tree and their hosts’ tree from Jane software. Black, bold lines (independent) represent the host phylogeny. Black, thin lines (dependent) correspond to the symbiont phylogeny. The events and costs considered were co-speciation (7), host-switching events (3), duplication followed by host-switching (2), and loss (3)

The Jane event-based reconstruction recovered seven potential co-speciation events between the host-insect and the symbiont—bacteria *Tremblaya* (Fig. 3). The reconstruction also recovered three host-switching events, two duplications followed by a host switch and three losses, for a total cost of 13.

### The mode of transovarial transmission of *Tremblaya phenacola* is identical in all species examined

**Fig. 4:**
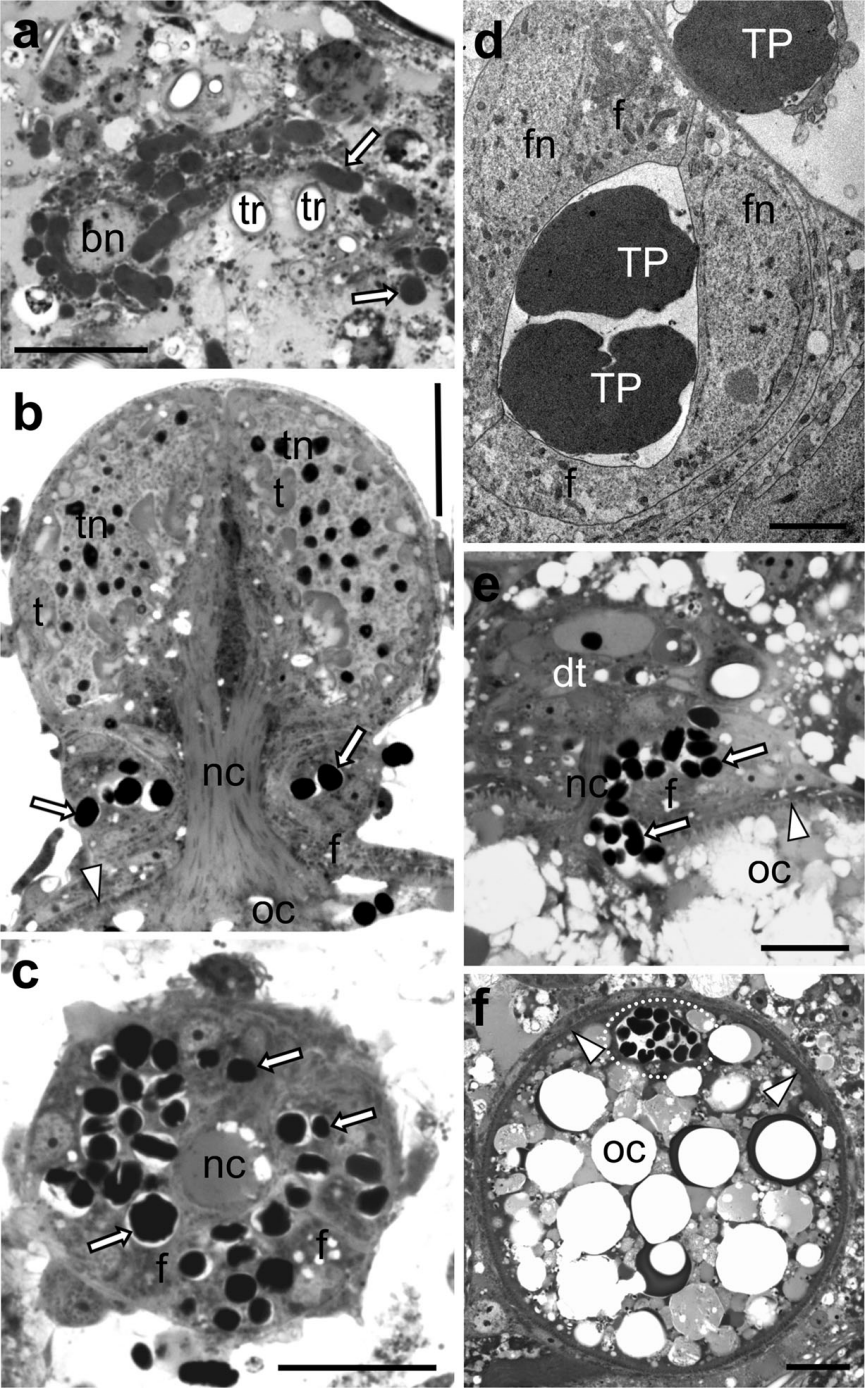
Transovarial transmission of bacteria *Tremblaya phenacola* between generations. **a***Coccura comari*. Bacteria *T. phenacola* (*white arrows*) leave the bacteriocytes during the initial stage of symbiont transmission. Bacteriocyte nucleus (*bn*); trachea (*tr*). LM, scale bar = 20 μm. **b***Phenacoccus aceris*. Longitudinal section through the ovariole. Symbiotic bacteria invade the neck region of the ovariole. Nutritive cord (*nc*); follicular cell (*f*); trophocyte (*t*); trophocyte nucleus (*tn*); oocyte (*oc*); bacterium *T. phenacola* (*white arrow*); egg envelopes (*white arrowhead*). LM, scale bar = 20 μm. **c***P. aceris*. Cross-section through the neck region of the ovariole. Note the bacteria *T. phenacola* (*white arrows*) migrating between follicular cells (*f*). Nutritive cord (*nc*). LM, scale bar = 20 μm. **d***Ceroputo pilosellae*. Fragment of the follicular epithelium surrounding the neck region of the ovariole. Symbionts migrate through the spaces between neighboring follicular cells (*f*). Bacterium *T. phenacola* (*TP*); nucleus of the follicular cell (*fn*). TEM, scale bar = 2 μm. **e***P. aceris*. The migration of symbiotic bacteria along the nutritive cord (*nc*) to the perivitelline space. Bacteria *T. phenacola* (*white arrows*); oocyte (*oc*); egg envelopes (*white arrowhead*); degenerating tropharium (*dt*). LM, scale bar = 20 μm. **f***C. pilosellae*. “Symbiont ball” (*encircled in white dotted line*) in the perivitelline space. Oocyte (*oc*); egg envelopes (*white arrowhead*). LM, scale bar = 20 μm

Histological analyses have shown that the *T. phenacola* of the insects examined is transovarially transmitted between generations (Fig. 4a–f). We observed that, in larvae and young females, bacteriocytes build large, compact bacteriomes (Fig. 1a), whereas in adult females these organs undergo disintegration (Fig. 1b). In consequence, individual bacteriocytes occur in the body cavity (Fig. 1b). In females that possess oocytes in the stage of late choriogenesis bacteria, *T. phenacola* leave the bacteriocytes (Fig. 4a) and migrate towards the ovaries. We observed that during the migration, bacteria stain more intensively with methylene blue and change shape from irregular to almost spherical (Fig. 4a). The infection takes place in the neck region of the ovariole (i.e., in the region between the tropharium and vitellarium—for further details concerning the organization of the ovariole of mealybugs, see (Szklarzewicz 1998)) and begins at the time that the tropharium is still connected with the oocyte developing in the vitellarium via the nutritive cord (Fig. 4b). At the beginning of transmission, symbionts migrate via spaces between neighboring follicular cells to the space between the follicular epithelium and the nutritive cord (Fig. 4b–d). During the next step of the transmission, bacteria *Tremblaya* move along the nutritive cord into the perivitelline space (the space between the oolemma and follicular epithelium) (Fig. 4e) where they gather to form a “symbiont ball” in the deep depression of the oolemma (Fig. 4f).

## Discussion

Mealybugs, like other phloem-feeding hemipterans, have established mutualistic relationships with bacteria (Buchner 1965; von Dohlen et al. 2001; Thao et al. 2002; Baumann and Baumann 2005; Gruwell et al. 2010; Gatehouse et al. 2011; Husnik et al. 2013; Koga et al. 2013; Lopez-Madrigal et al. 2015; Husnik and McCutcheon 2016; Szabo et al. 2017; Gil et al. 2018), which supplement their diet with essential amino acids and vitamins (Husnik et al. 2013; Husnik and McCutcheon 2016; Gil et al. 2018). Our molecular investigations have revealed that the Phenacoccinae mealybugs examined *Rhodania porifera*, *Phenacoccus aceris*, *Mirococcus clarus*, *Peliococcus calluneti*, *Ceroputo pilosellae*, and *Coccura comari* harbor betaproteobacterium *Tremblaya phenacola*. These results correspond with the previous studies concerning the symbiotic companions of Phenacoccinae mealybugs (Gruwell et al. 2010; Koga et al. 2013; Husnik and McCutcheon 2016; Gil et al. 2018). The bacteria *T. phenacola* were detected in almost all the representatives of the Phenacoccinae subfamily thus far examined, except mealybugs belonging to the genus *Rastrococcus* (Gruwell et al. 2010; Husnik et al. 2013). It should be stressed that the results of the molecular analyses of symbiotic systems of mealybugs confirmed the earlier observations of Buchner (1957) (see the “Introduction” section). Buchner (1957) investigated five species of the genus *Rastrococcus* from the island of Java and revealed that these mealybugs may be host to rod-shaped bacteria (*Rastrococcus spinosus*), yeast-like microorganisms (*Rastrococcus franseni*), or both (*Rastrococcus iceryoides*). More recently, Gruwell et al. (2010), using molecular approaches, revealed that bacteria which reside in mealybugs from the genus *Rastrococcus* belong to the phylum Bacteroidetes. Until now, there has been no information concerning the systematic affiliation of yeast-like microorganisms occurring in *Rastrococcus* species. In contrast to the members of Phenacoccinae subfamily, almost all the mealybugs from the Pseudococcinae subfamily live in symbiotic relationships with bacteria *T. princeps* and intra-*Tremblaya* various species of gammaproteobacteria (von Dohlen et al. 2001; Thao et al. 2002; Baumann and Baumann 2005; Koga et al. 2013; Lopez-Madrigal et al. 2015; Husnik and McCutcheon 2016; Szabo et al. 2017). Husnik and McCutcheon (2016) analyzed symbiotic systems of six species of Pseudococcinae mealybugs and revealed that in each of them, the cells of bacteria *Tremblaya* are colonized by different gammaproteobacteria. Due to the fact that these gammaproteobacterial symbionts exhibit large differences in genome size, these authors suggested that (1) the ancestor of extant Pseudococcinae was infected by a single gammaproteobacterium and (2) the observed diversity is the result of symbiont replacement. To our knowledge, there is only one exception to this rule—in myrmecophilous mealybugs from the genus *Hippeococcus* symbiotic microorganisms have not been detected despite the fact that they possess bacteriocytes (Buchner 1957). It is worth noting that in *Hippeococcus*, ovaries and embryos may begin to develop only when females have reached the nest of ants where they are then fed on a special juice rich in vitamins by the ants (Buchner 1957). This example clearly indicates that in a situation where mealybugs have unlimited access to nutritive feed, they may lose their symbionts.

In all the species analyzed, bacteria *T. phenacola* are localized in the insect’s cells—bacteriocytes. Such localization is characteristic for the long-term association between an insect and its symbiont, and it has been observed in the majority of scale insects (Buchner 1965; Szklarzewicz et al. 2006, 2018; Michalik et al. 2018a). However, some scale insects associated with bacteria (e.g., eriococcids *Acanthococcus aceris* and *Gossyparia spuria*) have not yet developed bacteriocytes, and, as a result, their symbionts occur in the fat body cells (Michalik et al. 2016). According to Michalik et al. (2016), the lack of bacteriocytes and occurrence of symbionts in the fat body cells may be an indication of the initial stage of symbiosis.

Taking into account the fact that symbionts are necessary for their proper growth and reproduction, insects have developed various modes of their transmission between generations (Buchner 1965; Kikuchi 2009; Szklarzewicz and Michalik 2017). In the majority of insects (e.g., hemipterans belonging to Sternorrhyncha and Auchenorrhyncha), symbionts are transovarially transmitted from mother to the offspring (see Buchner 1965; Szklarzewicz and Michalik 2017 for further details); however, in some, they may be transferred via milk-gland (tsetse fly), egg capsules, egg smearing, or acquisition from the environment in each generation (stinkbugs) (Kikuchi 2009). In all the examined Phenacoccinae mealybugs, bacteria *Tremblaya* invade the ovarioles in their neck region migrating to the perivitelline space via neighboring follicular cells. A similar mechanism of symbiont transmission was observed by Buchner (1957) in representatives of Phenacoccinae mealybugs of the genus *Rastrococcus.* In *C. pilosellae*, *C. comari*, *M. clarus*, *P. aceris*, *R. porifera*, and *P. calluneti***(**this study), as well as in *Rastrococcus* (Buchner 1957), bacteria and/or yeast-like microorganisms penetrate the follicular epithelium around the nutritive cord and then move to the perivitelline space where they assemble. The only difference is the form in which symbionts gather in the perivitelline space. In the forementioned Phenacoccinae (this study), they shape into a characteristic symbiont ball, whereas in *Rastrococcus*, they gather in the cap-shaped aggregation (Buchner 1957). Infection at the anterior pole of the oocyte (which is rather unique among insects) was also observed in Pseudococcidae mealybugs (von Dohlen et al. 2001), in the eriococcids *Greenisca brachypodii*, *A. aceris*, *G. spuria*, and in *Puto superbus* (Putoidae) (Michalik et al. 2016; Michalik et al. 2018a; Szklarzewicz et al. 2018). We observed that in the species examined, migrating symbionts change shape as well as stain more intensively in methylene blue than those in the bacteriocytes. The changes in shape and intensity of symbiont staining during migration towards the ovarioles have been described in several insect species, e.g., in scale insects from the Monophlebidae family (Szklarzewicz et al. 2006; Niżnik and Szklarzewicz 2007), in Deltocephalinae leafhoppers (Kobiałka et al. 2018), and in planthoppers (Michalik et al. 2018b). Michalik et al. (2018b) showed the unique transformation of the bacteria *Vidania* in the planthopper *Ommatidiotus dissimilis* (Fulgoromorpha), which drastically change shape from lobated in the bacteriome to almost spherical during migration.

Phylogenetic analysis based on sequences of the 16S rRNA gene of bacteria belonging to the genus *Tremblaya* has shown that bacteria *T. phenacola* of various species of Phenacoccinae, as well as bacteria *T. princeps* associated with Pseudococcinae, in relation to each other, constitute sister monophyletic groups (see Fig. 2). According to Gruwell et al. (2010), the infection of the ancestor of mealybugs by the bacteria *Tremblaya* took place before the splitting of Pseudococcidae family into two subfamilies and, from that time, they have co-evolved with their host insects independently. In Pseudococcinae, gammaproteobacteria have settled in bacteria *T. princeps* (von Dohlen et al. 2001; Lopez-Madrigal et al. 2015; Husnik and McCutcheon 2016; Szabo et al. 2017; Gil et al. 2018), whereas in some representatives of Phenacoccinae, bacteria *T. phenacola* have been replaced by other bacteria and/or yeast-like microorganisms, or even lost (Buchner 1957; Gruwell et al. 2010). The independent evolution of bacteria *T. princeps* and *T. phenacola* in the Pseudococcinae and Phenacoccinae subfamilies (respectively) was confirmed by Koga et al. (2013), who analyzed the symbiotic systems of two representatives of mealybugs residing on the same host plant—*Rhododendron pulchrum* (Ericaceae): *Crisicoccus azalea* (Pseudococcinae) and *Phenacoccus azalea* (Phenacoccinae). In spite of the fact that the insects examined have the same source of nutrients, they possess different symbionts—characteristic of Pseudococcinae and Phenacoccinae, respectively.

So far, co-speciation between scale insects and their symbionts was confirmed only in Pseudococcinae mealybugs (Thao et al. 2002; Baumann and Baumann 2005; Downie and Gullan 2005). Thao et al. (2002) have indicated that relationships among obligatory symbiont *T. princeps* may be useful in inferring the phylogeny of their hosts. Based on *Tremblaya* (16S-23S rRNA) and insect’s (18S-23S rRNA, cytB, EF-1α) genes, Baumann and Baumann (2005), as well as Downie and Gullan (2005), supported the previous conclusion that Pseudococcinae mealybugs co-evolved with bacteria *T. princeps*. However, in analyses carried out by Downie and Gullan (2005), the relationships between symbionts reflect the phylogeny of their host-insects only in 75%. A co-phylogenetic analysis on the basis of COI genes of mealybugs from the subfamily Phenacoccinae and 16S rRNA genes of their bacterial symbionts *T. phenacola* revealed their possible co-speciation relationships. Furthermore, more evolutionary events, i.e., host switching, loss, and duplication contributed to the evolution of Phenacoccinae species and their obligatory symbiont. However, in the case of certain species, the phylogenetic tree of symbionts is not concordant with the host phylogeny, most likely due to the fact that only one insect gene was used for the analysis. Our results, as well as the results of previous studies (Baumann and Baumann 2005; Downie and Gullan 2005), indicate that more detailed co-phylogenetic analyses, including a greater variety of the species of scale insects, as well as more insect genes, are needed.

## Electronic supplementary material

Fig. S1 **Bacteria*Tremblaya phenacola*in*Phenacoccus aceris*,*Ceroputo pilosellae*,*Coccura comari*,*Mirococcus clarus*,*Rhodania porifera*and*Peliococcus calunetti.*a, b***P. aceris*. Bacteriocyte nucleus (*bn*). a LM, scale bar = 20 μm. b TEM scale bar = 2 μm. **c, d***C. pilosellae*. Bacteriocyte nucleus (*bn*). c LM, scale bar = 20 μm. d TEM scale bar = 2 μm. **e, f***C. comari*. Bacteriocyte nucleus (*bn*). e LM, scale bar = 20 μm. f TEM scale bar = 2 μm. **g, h*M. clarus.*** g LM, scale bar = 20 μm. h TEM scale bar = 2 μm. **i, j***R. porifera*. Bacteriocyte nucleus (*bn*). i LM, scale bar = 20 μm. j TEM scale bar = 2 μm. **k, l***P. calunetti.* k LM, scale bar = 20 μm. l TEM scale bar = 2 μm (PNG 5872 kb)

High resolution image (TIF 49284 kb)
