# Supplementary figures and images for: Molecular characterization, ultrastructure, and transovarial transmission of Tremblaya phenacola in six mealybugs of the Phenacoccinae subfamily (Insecta, Hemiptera, Coccomorpha)
Source: Protoplasma. 2019 Jun 27;256(6):1597–608. doi: 10.1007/s00709-019-01405-y (PMC6820616; doi:10.1007/s00709-019-01405-y)

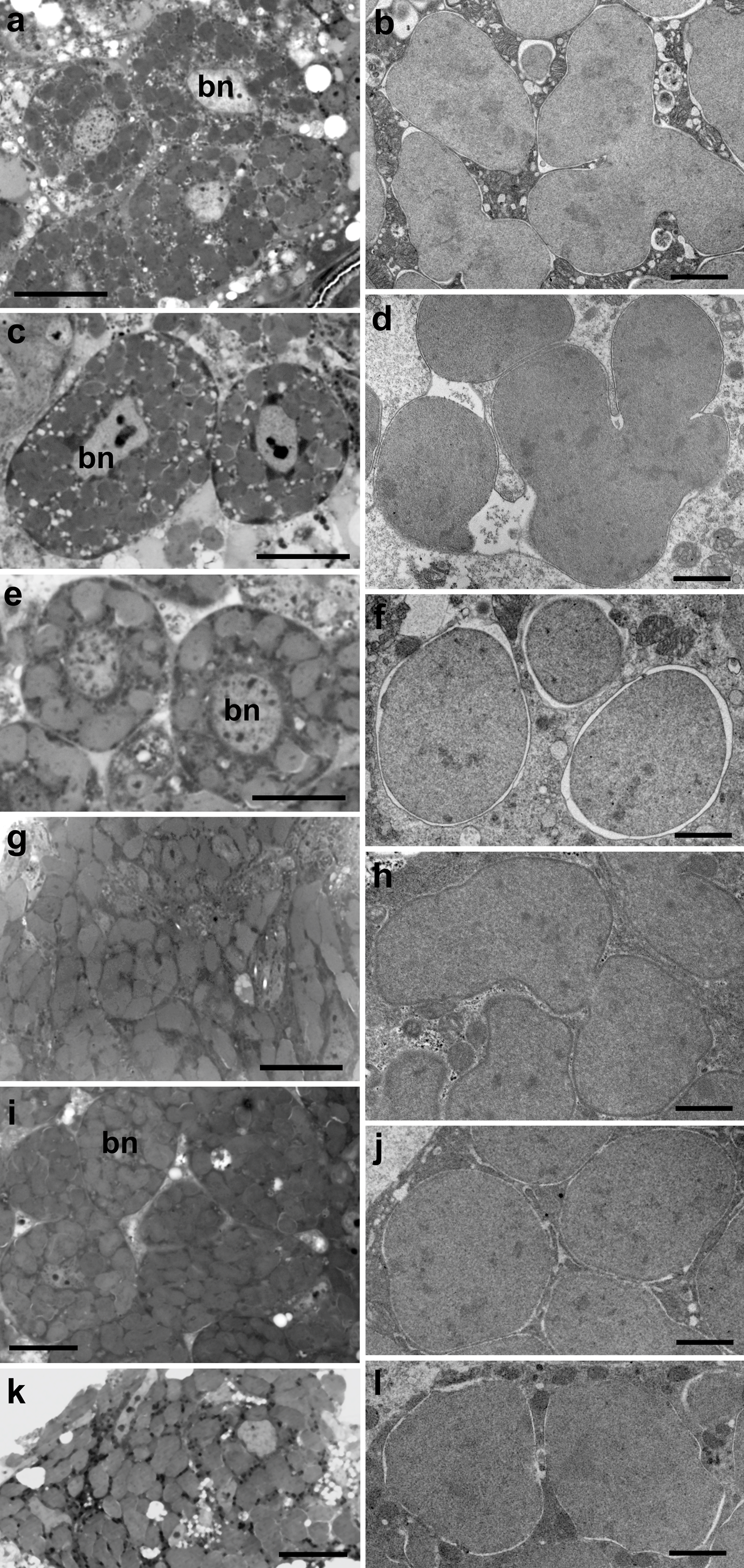

Supplement: Supplementary file 1 — BacteriaTremblaya phenacolainPhenacoccus aceris,Ceroputo pilosellae,Coccura comari,Mirococcus clarus,Rhodania poriferaandPeliococcus calunetti.a, bP. aceris. Bacteriocyte nucleus (bn). a LM, scale bar = 20 μm. b TEM scale bar = 2 μm. c, dC. pilosellae. Bacteriocyte nucleus (bn). c LM, scale bar = 20 μm. d TEM scale bar = 2 μm. e, fC. comari. Bacteriocyte nucleus (bn). e LM, scale bar = 20 μm. f TEM scale bar = 2 μm. g, hM. clarus. g LM, scale bar = 20 μm. h TEM scale bar = 2 μm. i, jR. porifera. Bacteriocyte nucleus (bn). i LM, scale bar = 20 μm. j TEM scale bar = 2 μm. k, lP. calunetti. k LM, scale bar = 20 μm. l TEM scale bar = 2 μm (PNG 5872 kb) [file 709_2019_1405_Fig5_ESM.png]

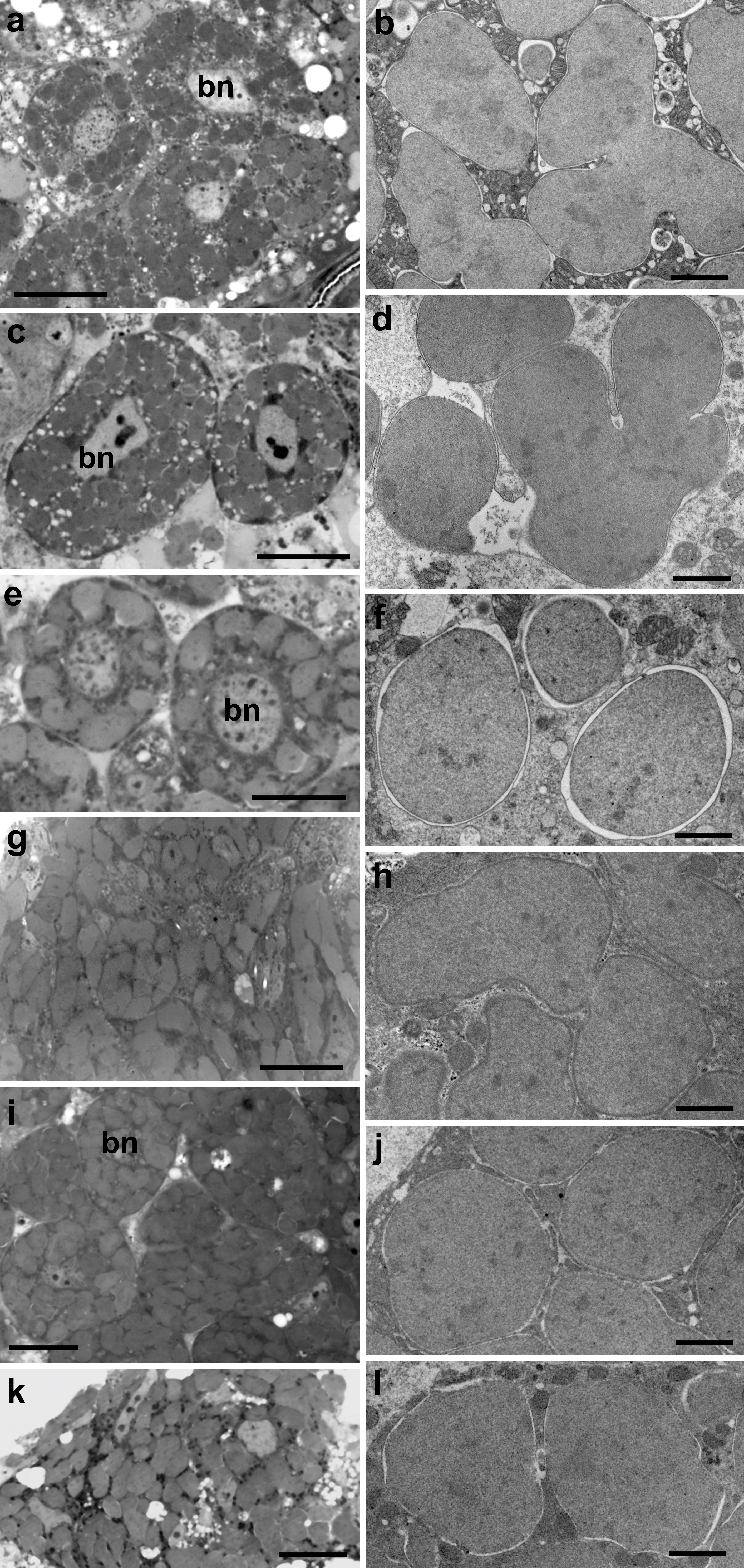

Supplement: Supplementary file 2 — High resolution image (TIF 49284 kb) [file 709_2019_1405_MOESM1_ESM.tif]
